# Rationale and Design of Khuzestan Vitamin D Deficiency Screening Program in Pregnancy: A Stratified Randomized Vitamin D Supplementation Controlled Trial

**DOI:** 10.2196/resprot.7159

**Published:** 2017-04-07

**Authors:** Maryam Rostami, Fahimeh Ramezani Tehrani, Masoumeh Simbar, Farhad Hosseinpanah, Hamid Alavi Majd

**Affiliations:** ^1^ Department of Reproductive Health and Midwifery Faculty of Nursing and Midwifery Shahid Beheshti University of Medical Sciences Tehran Islamic Republic Of Iran; ^2^ Reproductive Endocrinology Research Center Research Institute for Endocrine Sciences Shahid Beheshti University of Medical Sciences Tehran Islamic Republic Of Iran; ^3^ Obesity Research Center Research Institute for Endocrine Sciences Shahid Beheshti University of Medical Sciences Tehran Islamic Republic Of Iran; ^4^ Department of Biostatistics Faculty of Allied Medical Sciences Shahid Beheshti University of Medical Sciences Tehran Islamic Republic Of Iran

**Keywords:** vitamin D deficiency, pregnancy, clinical trial

## Abstract

**Background:**

Although there have been marked improvements in our understanding of vitamin D functions in different diseases, gaps on its role during pregnancy remain. Due to the lack of consensus on the most accurate marker of vitamin D deficiency during pregnancy and the optimal level of 25-hydroxyvitamin D, 25(OH)D, for its definition, vitamin D deficiency assessment during pregnancy is a complicated process. Besides, the optimal protocol for treatment of hypovitaminosis D and its effect on maternal and neonatal outcomes are still unclear.

**Objective:**

The aim of our study was to estimate the prevalence of vitamin D deficiency in the first trimester of pregnancy and to compare vitamin D screening strategy with no screening. Also, we intended to compare the effectiveness of various treatment regimens on maternal and neonatal outcomes in Masjed-Soleyman and Shushtar cities of Khuzestan province, Iran.

**Methods:**

This was a two-phase study. First, a population-based cross-sectional study was conducted; recruiting 1600 and 900 first trimester pregnant women from health centers of Masjed-Soleyman and Shushtar, respectively, using stratified multistage cluster sampling with probability proportional to size (PPS) method. Second, to assess the effect of screening strategy on maternal and neonatal outcomes, Masjed-Soleyman participants were assigned to a screening program versus Shushtar participants who became the nonscreening arm. Within the framework of the screening regimen, an 8-arm blind randomized clinical trial was undertaken to compare the effects of various treatment protocols. A total of 800 pregnant women with vitamin D deficiency were selected using simple random sampling from the 1600 individuals of Masjed-Soleyman as interventional groups. Serum concentrations of 25(OH)D were classified as: (1) severe deficient (<10ng/ml), (2) moderate deficient (10-20ng/ml), and (3) normal status (>20ng/ml). Those with severe and moderate deficiency were randomly divided into 4 subgroups and received vitamin D3 based on protocol and were followed until delivery. Data was analyzed according to the intention-to-treat principle.

**Results:**

Recruitment commenced in July, 2014, and as estimated, nearly 3.5 years is needed to complete the study. Results of this study will (1) provide reliable information regarding the prevalence of vitamin D deficiency during pregnancy using universal vitamin D screening approach and (2) determine the beneficial effects of universal screening and compare the various treatment protocols in terms of pregnancy outcomes.

**Conclusions:**

Since vitamin D deficiency is a prevalent disorder in pregnancy among Iranian population, this study will ensure creation of reliable evidence-based findings and will enable clinicians to better evaluate and treat vitamin D deficient pregnant women.

**Trial Registration:**

International Standard Randomized Controlled Trial Number (ISRCTN): 2014102519660N1; http://www.irct.ir/searchresult.php?keyword=&id=19660&number=1&prt=7805&total=10&m=1 (Archived by WebCite at http://www.webcitation.org/6p3lkqFdV)

## Introduction

### The Importance and Function of Vitamin D

Vitamin D as a fat-soluble steroid hormone is mainly synthesized by the skin on exposure to ultraviolet light and to a lesser extent can be ingested in the diet. It undergoes hydroxylation in the liver to produce an inactive supply form of 25-hydroxyvitamin D, 25(OH)D. Circulating 25(OH)D gets converted by renal 1-alpha-hydroxylase to the active form of 1,25-dihydroxyvitamin D3 which is the hormonally active form of vitamin D [1]. Since the half-life of 1,25(OH)D is short, circulating 25(OH)D is considered as the primary indicator of vitamin D status [2].

Vitamin D is best known for its effect on calcium hemostasis and bone metabolism [3], and plays a key role in cell differentiation, apoptosis, antiproliferation, immunosuppression, and antiinflammation in different body systems [4-6]. Recent growing evidence confirms its protective influence on the prevention of certain diseases like cancer [7], cardiovascular disorders [8], falls and fractures [9], autoimmunity [10], type-2 diabetes [11], and depression [12].

Although available data suggest the significant role for vitamin D deficiency in women’s reproductive health, the maternal and fetal function of vitamin D during pregnancy is not deeply recognized. One potential role of vitamin D during pregnancy is modulation of immune response [13]; however, evidence shows that it may also have functions on musculoskeletal [14] and cardiovascular systems [15] as well as neural development of the fetus [16]. Moreover, studies report a significant association between maternal vitamin D status and neonatal serum levels [17].

With respect to maternal vitamin D deficiency and adverse pregnancy outcomes, data are inconclusive and need further investigations. Trials have reported lower risk of preeclampsia and gestational diabetes in women receiving vitamin D supplementation compared with no intervention or placebo groups [18,19].

In terms of neonatal outcomes, data suggest reduced risk of preterm birth and low birth weight of less than 2500 grams in women who received vitamin D supplements. Interestingly, several observations have reported the influence of maternal vitamin D status beyond the postnatal period and have found its significant effect on childhood development and growth [20,21]. In regard with other pregnancy outcomes like mode of delivery, infant death, or neonatal biometric parameters, findings are not definite [22].

In recent years, the prevalence of vitamin D deficiency has increased and it is now recognized as a common global health concern and an ongoing pandemic [23]. Moreover, low levels of vitamin D during pregnancy have been reported in many populations worldwide, even in those with abundant sun exposure [24]. Previous findings have reported a varied prevalence of 18 to 84% for hypovitaminosis D during pregnancy, depending on the population studied [25-28]. Its prevalence among certain high-risk groups of pregnant women, particularly those with Middle Eastern origin like Iranian women (with low dietary vitamin D intake, high prevalence of pregestational obesity, and limited sun exposure) has been estimated to be approximately 60-80% [29-31]. However, due to the lack of consensus on the most accurate marker and test of vitamin D deficiency during pregnancy, and the optimal level of 25(OH)D for its definition, vitamin D deficiency assessment during pregnancy is a complicated process [32]. Furthermore, the comparison of vitamin D deficiency among different populations seems relatively troublesome due to the diversity of definitions, heterogeneity of the studied groups, or seasonal variations.

### Knowledge Gap

Although advising vitamin D consumption may prevent hypovitaminosis D in pregnant women, at this time there is insufficient evidence to support a recommendation for screening all pregnant women for vitamin D deficiency, and its beneficiary impact on pregnancy outcomes has not been well established. Besides, the optimal required dose during pregnancy is still undefined. In this respect, there is little population-based data available to quantify the treatment of vitamin D deficiency in pregnancy. In addition, the lack of high-quality evidence has hampered the implementation of vitamin D deficiency prevention programs and treatment protocols. Furthermore, there exists scarce data from Iranian population on the prevalence of gestational hypovitaminosis D.

### Clinical Endpoints

Primary endpoints are to find out the beneficiary impact of screening of pregnant women for vitamin D deficiency on pregnancy and neonatal outcomes and assessing the effect of supplementation with vitamin D on these outcomes. In the screening group, within the framework of randomized controlled trial, the endpoint was cord serum concentration of 25(OH)D to compare various doses, regimen, and routes for vitamin D supplementation in pregnancy.

In this regard, primary outcomes are gestational diabetes, preterm delivery, and pregnancy induced hypertension. Secondary outcomes are type of delivery and neonatal outcomes including birth weight (gr); head circumference (cm), height (cm); appearance, pulse, grimace, activity, respiration (APGAR) score 1, 5 minute, icterus, cord falling off time (day), serum calcium concentration (mg/dl), and cord levels of 25(OH)D (ng/ml).

### Research Questions

The questions are as follows: (1) How is the status of vitamin D among pregnant women in Khuzestan? (2) Is vitamin D deficiency related to adverse maternal and neonatal outcomes in pregnancy? (3) Does supplementation with vitamin D improve maternal and neonatal outcomes? (4) Is there any difference between various doses, regimen, and route for vitamin D supplementation in terms of pregnancy outcomes? (5) Taken altogether, is it reasonable to advise universal vitamin D deficiency screening program to detect and treat vitamin D deficiency in pregnancy?

## Methods

### Overall Study Design

This was a 2-phase population-based study. The first phase comprised a population-based cross-sectional study in which 1600 and 900 first trimester pregnant women attending health centers of Masjed-Soleyman and Shushtar, Khuzestan province, Iran were recruited using stratified multistage cluster sampling with probability proportional to size (PPS) method [33]. In the second phase of this study, within the framework of screening regimen, Masjed-Soleyman participants with vitamin D deficiency were assigned to a screening program versus Shushtar participants (as nonscreening arm) to assess the effectiveness of various treatment regimens. Pregnancy outcomes including preterm delivery, miscarriage, preeclampsia, gestational diabetes, and type of delivery and neonatal outcomes including birth weight (gr), head circumference (cm), height (cm), APGAR score 1, 5 minute, icterus, cord falling off time (day), serum calcium (mg/dl) level, and cord concentration of 25(OH)D (ng/ml) were recorded.

Shushtar participants (as nonscreening arm) served as controls and were followed till delivery and any adverse pregnancy outcomes were assessed.

### The First Phase

In the first phase, pregnant women attending prenatal care centers of urban areas of Masjed-Soleyman and Shushtar were recruited from July 1-September 31, 2014. Due to the blinding purposes and to avoid contacts among study subjects [34], we chose 2 cities with similar cultural, geographic, nutritional habits, and sun exposure conditions; one of these cities was assigned to intervention.

Masjed-Soleyman County is in the northeast of Khuzestan province. Its area is 9/6327 km^2^ with a population of 103,369 people with Persian ethnicity. This is a sunny region with a hot and humid climate. Its altitude is 260 meters above sea level. In terms of geographical location, it is between 31°59′ E longitude and 49°17′ N latitude. Shushtar County is in the north of Khuzestan. Its area is 2436 km^2^ with a population of 192,361 people with Persian ethnicity. The climate is similar to Masjed-Soleyman. Its altitude is 150 meters above sea level. In terms of geographical location, it is between 48°20′ E longitude and 32°30′ N latitude.

### Blood Assessments

Upon enrollment into the study, a fasting blood sample was obtained from all participants. Separated serum samples by centrifugation were transferred to the central laboratory of Masjed-Soleyman (cold chain was kept during storage and transfer processes). The serum samples of participants in Shushtar were stored and kept frozen at -80°C until assayed at the end of the study, but at that time the serum concentration of vitamin D of Masjed-Soleyman participants were promptly measured. Based on 25(OH)D levels, mothers were divided as severe deficient (<10ng/ml), moderate deficient (10-20ng/ml), and normal status (>20ng/ml) [1]. The scale to express 25(OH)D status results was according to ng/ml; each 1 ng/ml is equal to 2.496 nmol/L.

**Figure 1:**
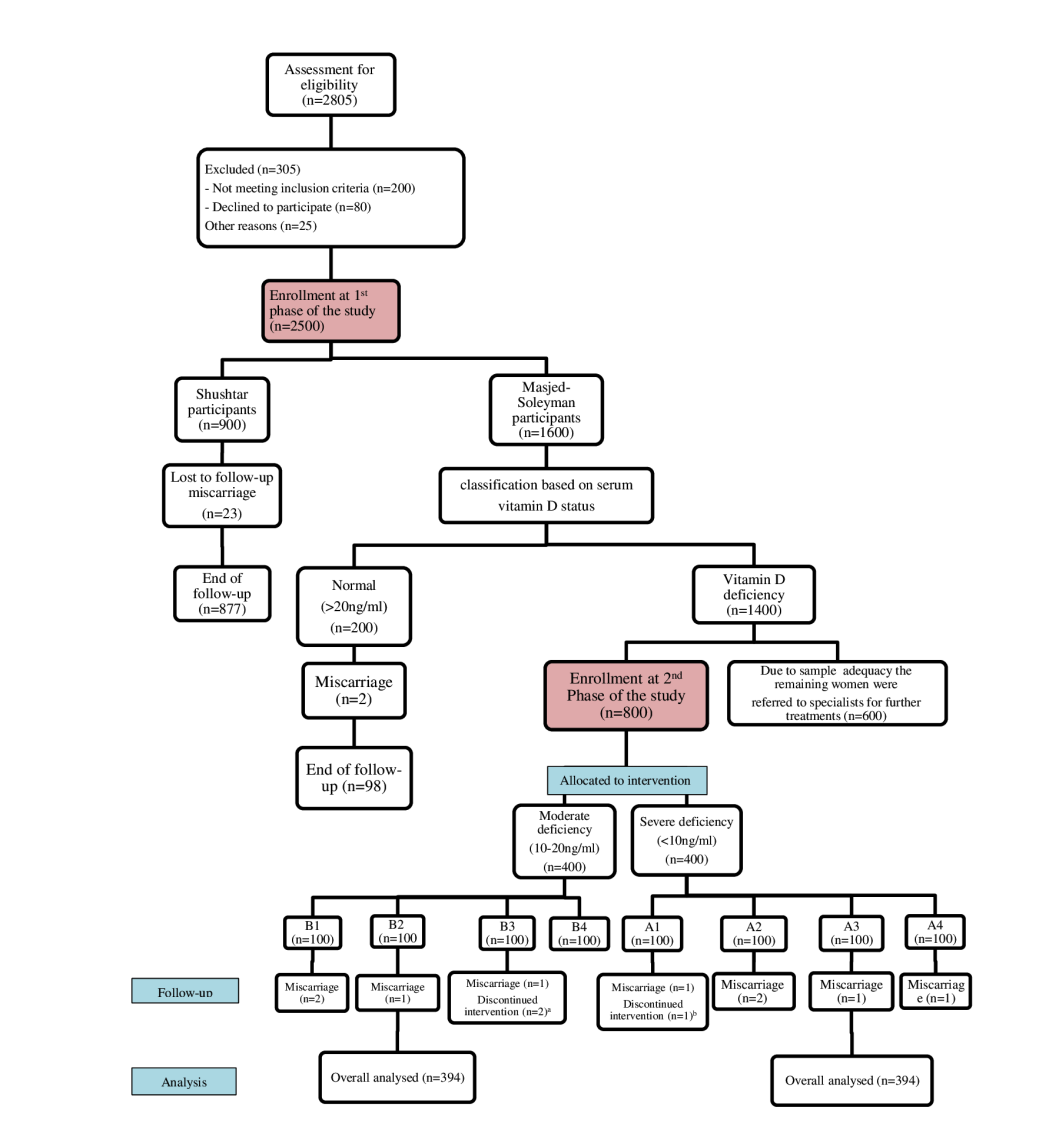
The study participant flow diagram. (a) Discontinued intervention due to car accident and humerus injury (n=1) and dislike to continue vitamin D3 supplementation weeks after consumption (n=1). (b) Discontinued intervention due to husband’s death and subsequent mental problems.

Those participants from Shushtar and those with serum vitamin D>20 ng/ml from Masjed-Soleyman served as controls and were followed until delivery. Subjects with severe and moderate vitamin D deficiency from Masjed-Soleyman were selected for the second phase of the study and were randomly allocated to one of the treatment modalities presented in Figure 1.

### Subject Recruitment and Eligibility Criteria

Pregnant women, aged 18-40 years, were eligible if they had gestational age <14 weeks based on last menstrual period or obstetrical estimation, singleton pregnancy, and had planned to receive ongoing prenatal and delivery in the Masjed-Soleiman. Participants were excluded if they consumed multivitamins containing more than 400 international unit (IU) per day of vitamin D3; used anticonvulsants; and had history of chronic diseases like diabetes, hypertension, renal dysfunction, liver diseases, and complicated medical or obstetrical history.

### Data Collection and Quality Control Procedures

At first, midwives responsible for prenatal care in the selected health centers were invited to attend a workshop designed for explaining the study objectives and procedure. Thereafter, first trimester pregnant women seeking maternity care during their first routine prenatal visit were invited to participate in the study after providing a detailed explanation of the study procedure; they signed a written informed consent during recruitment covering all trial procedures and data collection. For gestational age calculation, the first day of the last menstrual cycle (LMP) for women with regular cycles and ultrasonography for those with irregular cycles or those who could not precisely recall their LMP was used. Pregnant women received prenatal care and each adverse pregnancy outcome was managed according to the standard guidelines. At enrollment, for each participant a questionnaire that included information on sociodemographic, anthropomorphic, behavioral, and reproductive characteristics was completed by a trained interviewer.

Weight was measured with minimum clothing to the nearest 100 grams. Height was measured with a tape measure in standing position with normal posture of shoulders. Body mass index was calculated by dividing weight (kg) on height (m^2^). Systolic and diastolic blood pressures (SBP and DBP) were measured twice in a sitting position with a standard mercury sphygmomanometer after a 15-min rest and the mean of the 2 measurements was considered as SBP or DBP.

A fasting blood sample (5cc) was taken by venipuncture from the antecubital vein. Plasma was then separated by centrifugation (1500g, 10 min, 4˚C) in the central laboratory of Masjed-Soleyman and stored at −20˚C until analysis at the end of the week. Serum concentrations of 25(OH)D for the participants of Masjed-Soleyman were measured immediately, and subsequently their vitamin D status was determined (Table 1). The samples in Shushtar laboratory were stored at −80˚C until the end of the study; finally their serum concentration of vitamin D was measured.

Plasma 25(OH) vitamin D3 was assessed using enzyme-linked immunosorbent assay (ELISA) method and a kit of Immunodiagnostics Systems Ltd (IDS Ltd) by Auto Analyzer (Human Corporation, Germany). The sensitivity of the test was 5nmol/L and the intra and interassay coefficient of variations (CVs) were 3.37% and 3.891%, respectively. Calibration of the instruments was done as per the manufacturer's instructions and validation studies were done prior to the test. All measurements were done according to the standard operating procedures and samples were analyzed by a single technician using the same equipment throughout the study in a reference laboratory.

### The Second Phase

In the second phase of the study, to assess the effect of screening strategy on maternal and neonatal outcomes, Masjed-Soleyman participants were assigned to a screening program versus Shushtar participants acting as the nonscreening arm. Within the framework of the screening regimen, an 8-arm blind randomized clinical trial was undertaken to compare the effects of various treatment protocols. Due to the cost and complexity of the process, 800 pregnant women with vitamin D deficiency from Masjed-Soleyman were randomly allocated to 1 of the designed intervention programs according to the study Figure 1. The remaining women with vitamin D deficiency were referred to specialists for further treatments. Participants of Shushtar did not receive any vitamin D supplementation. However, the comparison of the basic confounders between the initial recruited sample in Masjed-Solayman and the women allocated to intervention indicated no statistically significant difference and hence no selection bias occurred during the allocation of treatment (Table 4).

### Sample Size Calculation

**Figure 2:**
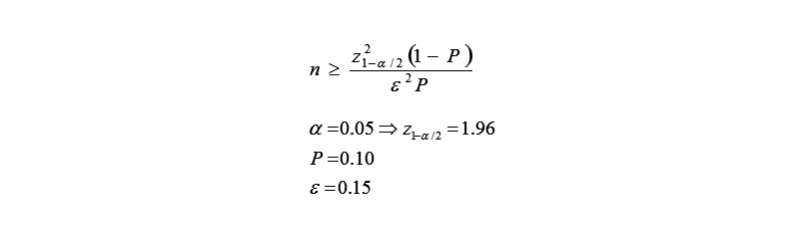
Sample estimation formula for the first phase of the study.

A cluster sampling method with PPS procedure was assigned. Sample size was calculated in screening group (Masjed-Soleyman) using the following formula (Figure 2) and assumption, resulting in 1537 subjects.

The same steps (except for ε=0.2) were used for calculation of sample size in no screening group (Shushtar), resulting in 900 subjects. Using the cluster sampling method, 1600 and 900 first trimester mothers were selected from among those receiving prenatal care in health centers in urban regions of Masjed-Soleyman and Shushtar, respectively.

Since the prevalence of the specified event is untreated or unrecognized and the number of people who have the risk factor (P) is high in the population, consequently, sample size needed for screening is considered sufficient. In other words, we define a correction coefficient by (1/P) for estimated sample size (n), and then we expect n (1/P) number of people to find the 0.15 difference with 5% significance level.

**Figure 3:**
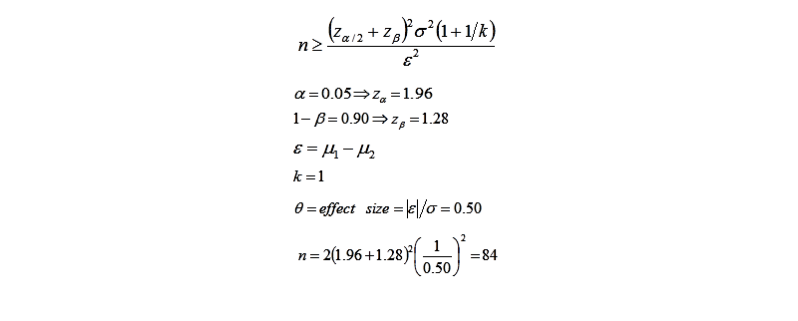
Sample estimation formula for the second phase of the study.

To compare different regimens used for vitamin D deficiency in the screening group, the sample size in each group was calculated based on the following formula (Figure 3) and assumption:

Considering loss to follow up of 10%, a total number of 100 in each study group was considered adequate.

### Randomization and Blinding

Subjects in each group of severe or moderate deficiencies were randomly divided into 4 subgroups using permuted block randomization by a biostatistician to achieve balance across treatment groups. The number of subjects per block was 8. Sealed opaque envelopes were assigned to each subject by a research assistant not associated in the trial. The dedicated study midwife treating the females, who did not participate in any subsequent phases of the study, was the only person who knew the group each patient belonged to (single blinded). Masking to treatment allocation was not possible and only those health care workers who determined pregnancy outcomes were blinded to treatment allocation.

### Intervention

The participants from Masjed-Soleyman entering the second phase of the study received vitamin D as follows.

#### Women With Severe Vitamin D Deficiency (Group A)

Group A1: Subjects were treated with 50,000 IU of oral vitD3 weekly for a total duration of 12 weeks.

Group A2: Subjects were treated with 50,000 IU of oral vitD3 weekly for a total duration of 12 weeks and then were on monthly maintenance dose of 50,000 IU vitD3 until delivery.

GroupA3: Subjects were treated with intramuscular administration of 300,000 IU vitD3; 2 doses for 6 weeks.

GroupA4: Subjects were treated with Intramuscular administration of 300,000 IU vitD3; 2 doses for 6 weeks and then were on monthly maintenance dose of 50,000 IU vitD3 until delivery.

#### Women With Moderate Vitamin D Deficiency (Group B)

Group B1: Subjects were treated with 50,000 IU oral vitD3 weekly for a total duration of 6 weeks.

Group B2: Subjects were treated with 50,000 IU oral vitD3 weekly for a total duration of 6 weeks and then were on monthly maintenance dose of 50,000 IU vitD3 until delivery.

GroupB3: Subjects were treated with a single dose of intramuscular administration of 300,000 IU vitD3.

GroupA4: Subjects were treated with a single dose of Intramuscular administration of 300,000 IU vitD3 and then were on monthly maintenance dose of 50,000 IU vitD3 until delivery.

Participants were advised to inform the health care provider about any adverse side effect. A fasting blood sample was obtained 3 months after termination of the intervention for those without maintenance vitamin D therapy (groups A1, A3, B1, and B3) and for all those in other groups at delivery. Also, a cord blood sample was obtained from participants. Separated serum samples after centrifugation were transferred to the central laboratory of Masjed-Soleyman. Samples were stored at −80°C and serum concentrations of vitamin D3 were measured at the end of the study.

#### Women With Normal Status (Group C)

Subjects in this group and the pregnant women recruited from Shushtar city comprised our controls. They had access to standard prenatal care and were followed until delivery. In the case of any adverse outcome, it was managed according to the perinatal guidelines. Blood sampling from subjects in this group and those from Shushtar was obtained at the first visit. Similar to others groups, another sample was obtained from mothers and umbilical cord at the time of delivery.

### Source of Vitamin D

Oral vitamin D3 50 000 U or cholecalciferol tablets were manufactured by Roche Pharmaceutical, Tehran, Iran and dispersed in Iran by Zahravi, Tehran, Iran. Intramuscular D3 injection of 1-ml ampoule, 300 000 IU/ml in sesame oil, was manufactured by Caspian Pharmaceutical, Iran.

### Adherence to Medication Regimen

Adherence to the supplementation regimen was measured by maternal self-report and pill counts at each prenatal visit. The number of pills returned was divided by the expected number of pills that would have been taken to create a percentage that indicates the adherence of medication regimen. The measures were used to make an average adherence for each subject [35].

### Statistical Analysis

Data will be expressed as mean (standard deviation, SD) for normally distributed variables and frequency (%) or median (interquartile range, IQR) for categorical and or nonnormal variables. Continuous variables are checked for normality using the one sample Kolmogorov-Smirnov test. Distribution of variables between the 2 groups is compared using *t* test or Mann-Whitney nonparametric test in case of normality violation and reported as mean (SD). Categorical variables are compared using Pearson and chi-square tests. An intention-to-treat analysis of the results is used. Generalized linear models will be used to assess the relationships between outcomes and exposures (here vitamin D3 supplementation intervention) adjusted by appropriate covariates. Analyses will be performed using the software SPSS version 19 (SPSS Inc). *P* value of less than .05 is considered as significance level.

**Table 1:** The steps of study analysis.

Proceedings		Stage
Obtaining written inform consent form and completing the questionnaire		Recruitment and completion of the questionnaire
Determining groups according to their status of vitamin DCollection of the laboratory samples Obtaining fasting blood samples. Assessment of serum concentration of vitamin D in Masjed-Solayman immediately and storing Shustar samples at −80°C		Classification
Obtaining fasting blood samples 3 months after termination of the intervention for those without maintenance vitamin D therapy (groups A1, A3, B1, and B3) and for all women at delivery time. Obtaining cord blood sample from all participantsStart of intervention Prescription of vitamin D according to various protocols (A1-3 and B1-3) for intervention groups		Replication of laboratory samples
Follow-up of pregnancy outcomes and neonatal calcium and vitamin D status		Follow-up
	
	

### Definition of Study Outcomes

In this study the primary outcome was vitamin D deficiency measured as serum level of 25(OH)D and was categorized as: (1) severe deficient (<10ng/ml), (2) moderate deficient, (10-20ng/ml), and (3) normal status (>20ng/ml) [36].

Our secondary outcomes were as follows: Preterm delivery was considered as birth at less than 37 completed weeks of gestation [37]. Miscarriage was referred to as pregnancy loss prior to 20 weeks from LMP [38]. Preeclampsia was defined as systolic blood pressure >140 mmHg or diastolic blood pressure ≥90 mmHg and 24-hour proteinuria ≥0.3 g, started at >20 weeks [39]. Gestational diabetes mellitus referred to glucose intolerance first detected during pregnancy and was diagnosed according to the *International Association of the Diabetes and Pregnancy Study Groups (IADPSG)* criteria [40]. Premature rupture of membrane was considered as rupture of the fetal membranes before the onset of labor regardless of gestational age [41]. By type of delivery, we meant cesarean section or vaginal delivery. Birth weight (gr) was defined as weight at birth irrespective of gestational age [42].

APGAR score comprises 5 components including *appearance, pulse, grimace, activity, and respiration*, each of which is given a score of 0-2 and reported at 1 and 5 minutes after birth [43]. Neonatal icterus was defined as the yellowish discoloration of skin and sclera by bilirubin, usually first noted in the face and then the body [44]. Cord falling off time (day) is the separation time of remained umbilical cord after birth [45].

### Season

Blood samples were obtained in spring (April-May), summer (July-September), fall (October-December), and winter (February-March).

### Safety Considerations

It has been suggested that vitamin D supplementation can safely be utilized in pregnancy [22]. Furthermore, our study participants who received treatment had vitamin D deficiency and hence, the risk of hypervitaminosis D was reduced to almost zero. Also, during the screening program, any medical condition or probable side effects of vitamin D detected were promptly recorded and discussed by a qualified medical specialist involved in the study.

### Approval and Ethical considerations

The protocol was approved by the medical ethics committee of the research institute for endocrine sciences of Shahid Beheshti University of Medical Sciences (10ECRIES25/10/92) (Trial Registration: IRCT2014102519660N1). The reviewers’ comments and acceptance letter are provided (see Multimedia Appendices 1-3). Participants were required to sign a written informed consent at recruitment covering all trial procedures and data collection. It was emphasized that participation in the study was voluntary and they were free to withdraw from the study at any time.

## Results

Recruitment commenced in July, 2014 and as estimated, nearly 3.5 years is needed to complete the study. Currently, this study is in the stage of analyzing data.

The baseline characteristics of the study participants are shown in Tables 2 and 3, which indicate no significant difference between the participants of the 2 cities.

**Table 2:** Baseline characteristics of study participants, overall and by study sites.

Characteristics	Masjed-Solayman n=900	Shushtar n=900	Overall n=1800	*P* value^b^
Age (year)	29 (25-32)^a^	29 (25-32)	29 (25-32)	.63
Marriage age (year)	20 (18-22)	19 (17-23)	19 (17-22)	.08
First delivery age (year)	20 (18-22)	20 (18-21)	20 (18-22)	.57
First pregnancy age (year)	21 (19-24)	20 (18-24)	21 (19-24)	.08
Pregnancy week	10 (9-12)	10 (9-12)	9 (10-12)	.07
Gravity	2 (1-3)	2 (1-3)	2 (1-3)	.95
Parity	1 (0-2)	1 (0-2)	1 (0-2)	.86
Number of abortions	0 (0-0)	0 (0-0)	0 (0-0)	.96
Number of children	1 (0-2)	1 (0-2)	1 (0-2)	.79
Vitamin D4 (ng/ml)	11 (7-16)	11 (7-16)	11 (7-16)	.96
SBP (6-10w) (mg/dl)	115 (110-120)	120 (110-120)	120 (110-120)	.87
DPB (6-10w) (mg/dl)	70 (60-70)	70 (70-70)	70 (60-70)	.65
Maternal weight (6-10 w)	65 (59-70)	64 (59-70)	64 (59-70)	.98

^a^Median (interquartile range).

^b^*P* value obtained from independent samples distribution nonparametric Mann-Whitney test (the significance level is <.05).

**Table 3:** Baseline qualitative characteristics of the study participants, overall and by study site.

Participant characteristics		Masjed Solayman		Shushtar		Overall			
		Frequency (n)	Percentage (%)	Frequency (n)	Percentage (%)	Frequency (n)	Percentage (%)	^a^*P* value	
**Education**										
	Illiterate	37	4.10	17	1.90	54	3.00	.07
	Primary	132	14.70	135	15.00	267	14.80	
	Guidance school	218	24.20	240	26.70	458	25.40	
	High school	319	35.40	313	34.80	632	35.10	
	Academic	194	21.60	195	21.70	389	21.60		
	Total	900	100.00	900	100.00	1800	100.00	
**Spouse education**									
	Illiterate	33	3.70	24	2.70	57	3.20	.01^a^
	Primary	136	15.10	144	16.00	280	15.60	
	Guidance school	178	19.80	207	23.00	385	21.40	
	High school	249	27.70	190	21.10	439	24.40	
	Academic	304	33.80	335	37.20	639	35.50	
	Total	900	100.00	900	100.00	1800	100.00	
**Spouse occupation**									
	Worker	377	41.90	332	36.90	709	39.40	.08
	Clerk	205	22.80	213	23.70	418	23.20	
	Self-employed	318	35.30	355	39.40	673	37.40	
	Total	900	100.00	900	100.00	1800	100.00	
**Accommodation**									
	Apartment	431	47.90	371	41.20	802	44.60	.004^a^
	House	469	52.10	529	58.80	998	55.40	
	Total	900	100.00	900	100.00	1800	100.00	
**Sun exposure**										
	<5	319	35.40	264	29.30	583	32.40	.001^a^
	May-15	383	42.60	282	31.30	665	36.90	
	15-30	161	17.90	235	26.10	396	22.00	
	>30	37	4.10	119	13.20	156	8.70	
	Total	900	100.00	900	100.00	1800	100.00	
**Cream**									
	Mostly	310	34.40	174	19.30	484	26.90	.001^a^
	Sometimes	321	35.60	386	42.90	707	39.20	
	Always	269	29.90	340	37.80	609	33.80	
	Total	900	100.00	900	100.00	1800	100.00	

**Sun glass**									
	Yes	326	36.20	297	33.00	623	34.60	.15
	No	574	63.80	603	67.00	1177	65.40	
	Total	900	100.00	900	100.00	1800	100.00	
**Gloves**									
	Yes	193	21.40	143	15.90	336	18.70	.002^a^
	No	707	78.60	757	84.10	1464	81.30
	Total	900	100.00	900	100.00	1800	100.00	

**Veil**									
	Chador	328	36.40	502	55.80	830	46.10	.001^a^
	Uniform	572	63.60	398	44.20	970	53.90	
	Total	900	100.00	900	100.00	1800	100.00		
**Spouse smoke**									
	Yes	181	20.10	163	18.10	344	19.10	.28
	No	719	79.90	737	81.90	1456	80.90	
	Total	900	100	900	100.00	1800	100.00	
**Occupation**										
	Worker	14	1.60	21	2.30	35	1.90	.46
	Clerk	135	15.00	119	13.20	254	14.10	
	Self-employed	111	12.30	117	13.00	228	12.70	
	Housewife	640	71.10	643	71.40	1283	71.30	
	Total	900	100.00	900	100.00	1800	100.00	

^a^The chi-square statistic is significant at the .05 level.

^b^Fisher exact test results is significant at the .05 level.

For the second phase of the study, as statistically was calculated, we needed 800 women, therefore the remaining women were referred to a specialist for further treatments. However, in terms of baseline features except for vitamin D levels, there was no significant difference between the initial sample in Masjed-Soleyman and those who were allocated to intervention (Table 4). The difference for vitamin D is ignorable though.

**Table 4:** Comparison of baseline characteristics between the initial sample of Masjed-Soleyman participants and those allocated to intervention.

Baseline variables	Initial sample (n=1581)	Allocated for intervention (n=900)	*P* value^a^
Age (year)	29 (25-32)^b^	29 (25-32)	.77
First pregnancy age (year)	20 (18-22)	20 (18-22)	.94
First delivery age (year)	20 (18-22)	20 (18-22)	.73
Gestational age (week)	10 (9-12)	10 (9-12)	.97
Vitamin D	11.9 (8-17)	11.2 (7-16)	.01
Number of children	^b^1 (0-2)	1 (0-2)	.67
Gravity	2 (1-3)	2 (1-3)	.62
Parity	1 (0-2)	1 (0-2)	.47
Number of abortions	0 (0-0)	0 (0-0)	.94

^a^*P* value obtained from nonparametric Mann-Whitney test for nonnormal variables.

^b^Median (interquartile range, Q1-Q3).

## Discussion

### Principal Findings

Vitamin D deficiency is one of the most prevalent disorders among mothers and children [26]. Although in the past few decades, early diagnosis of vitamin D deficiency and availability of supplementations and treatment modalities have improved the deficiency condition; hypovitaminosis D still exists as a major public health concern associated with significant morbidities in a variety of countries [23]. Unfortunately, among Iranian women who have sunlight exposure deprivation and inadequate dietary vitamin D intake, it is highly prevalent. Reports indicate that 86% of Iranian pregnant women and 75% of their newborns suffer from vitamin D deficiency [46].

Despite the safety of maternal supplementation in preventing vitamin D deficiency during pregnancy, at the moment there is no certain preventive or interventive strategy to ensure maternal vitamin D sufficiency. Therefore, the effect of vitamin D supplementation in pregnant women remains an arguable problem.

In a double-blind randomized clinical trial, Hollis et al [47] determined the safety and efficacy of vitamin D supplementation in pregnant women. In that study, 350 first trimester pregnant women received 400, 2000, or 4000 IU of vitamin D per day until delivery. The relative risk (RR) of vitamin D deficiency was significantly different between the groups receiving 2000 and 400 IU (RR=1.52, 95% CI 1.24-1.86) and the groups receiving the 4000 and 400 IU (RR=1.60, 95% CI 1.32-1.95). Difference was not significant between 4000 and 2000 IU groups (RR=1.06, 95% CI 0.93-1.19). They concluded that vitamin D supplementation of 4000 IU/d for pregnant women is safe and most effective in achieving sufficiency in all women and their neonates, irrespective of race and ethnicity. The optimization of 25(OH)D and 1,25(OH)_2_ D levels was attained without any evidence of hypervitaminosis or increase in adverse side effects during pregnancy.

In a recent study, Mojibian et al investigated the effects of 50,000 IU of vitamin D every 2 weeks supplementation on the incidence of maternal and neonatal outcomes and compared it with the women who received 400 IU vitamin D per day. They reported that there were no differences in the incidence of maternal outcomes (preeclampsia, gestational hypertension, preterm labor, and low birth weight) and anthropometric measures in neonates between two groups except for gestational diabetes mellitus (GDM), which was significantly lower in the first group. They concluded that vitamin D supplementation with a dose of 50,000 IU vitamin D every 2 weeks could decrease the incidence of GDM [48]. In an accordant study, Rodda et al conducted an open-label randomized controlled trial and reported the preventive effects of vitamin D supplementation in neonatal deficiency among deficient mothers [49].

Nevertheless, in a systematic review by Harvey et al [20], authors reported insufficient evidence to support clinical recommendations regarding vitamin D supplementation in pregnancy. They concluded that although there may be a relationship between maternal 25(OH)D levels with newborn birth weight and bone mass, there is a risk of bias and therefore further randomized clinical trials are needed. Another recent systematic review by De-Regil et al [22] revealed that supplementing pregnant women with vitamin D in a single or continued dose increases serum 25(OH)D at term and may reduce the risk of preeclampsia, low birth weight, and preterm birth. However, the evidence on whether vitamin D supplementation should be given as a part of routine antenatal care to all women to improve maternal and infant outcomes remains unclear.

At present, novel investigations, specifically, randomized controlled trials are needed to assess the optimal vitamin D requirements of pregnant women particularly in high-risk populations with inadequate sunlight exposure. The present survey will address an attempt to update the optimal strategies on treatment or supplementation of vitamin D deficiency during pregnancy.

### Limitations

However, this study is subject to some limitations. First, conducting the study in urban regions restricts its generalizability to rural areas. Second, some pregnancies may be aborted prior to entering the study and hence it could decrease our power to interpret the results of miscarriage outcome. Third, the potential number of our lost to follow-up cases may be another limitation. Finally, fourth is the concern of vitamin D consumption or lack of adherence to the treatment regimen among the intervention groups.

Overall, the results of this study regarding vitamin D deficiency prevalence and optimal supplementation and treatment methods during pregnancy will empower clinicians with novel recommendations in patient decision-making.

## Appendix

Multimedia Appendix 1 Reviewers' comments.

Multimedia Appendix 2 Response to reviewers.

Multimedia Appendix 3 Acceptance letter.

Multimedia Appendix 4 CONSORT-eHEALTH (V 1.6.1) - submission/publication form.

## Abbreviations

25(OH)D: 25-hydroxyvitamin D
APGAR: appearance, pulse, grimace, activity, respiration
DBP: diastolic blood pressure
GDM: gestational diabetes mellitus
IQR: interquartile range
IU: international unit
RR: relative risk
SBP: systolic blood pressure
SD: standard deviation
