# Supplementary material for: Rationale and Design of Khuzestan Vitamin D Deficiency Screening Program in Pregnancy: A Stratified Randomized Vitamin D Supplementation Controlled Trial
Source: JMIR Res Protoc. 2017 Apr 7;6(4):e54. doi: 10.2196/resprot.7159 (PMC5400884; doi:10.2196/resprot.7159)
Supplement: Multimedia Appendix 4 [file resprot_v6i4e54_app4.pdf]

CONSORT 2010 checklist of information to include when reporting a randomised trial*

Title: Khuzestan vitamin D deficiency screening program in pregnancy: a stratified randomized vitamin D supplementation controlled trial; Rationale and Design

Section/Topic	Item No	Checklist item	Reported on page No
**Title and abstract**			
	1a	Identification as a randomised trial in the title	P.1
	1b	Structured summary of trial design, methods, results, and conclusions (for specific guidance see CONSORT for abstracts)	P.2
**Introduction**			
Background and objectives	2a	Scientific background and explanation of rationale	PP.4 and 5
	2b	Specific objectives or hypotheses	P.5
**Methods**			
Trial design	3a	Description of trial design (such as parallel, factorial) including allocation ratio	P.7,9,13
	3b	Important changes to methods after trial commencement (such as eligibility criteria), with reasons	Not applicable
Participants	4a	Eligibility criteria for participants	P.11
	4b	Settings and locations where the data were collected	PP. 7 and 11
Interventions	5	The interventions for each group with sufficient details to allow replication, including how and when they were actually administered	PP. 14 and 15
Outcomes	6a	Completely defined pre-specified primary and secondary outcome measures, including how and when they were assessed	PP. 16, 17
	6b	Any changes to trial outcomes after the trial commenced, with reasons	Not applicable
Sample size	7a	How sample size was determined	PP. 12, 13

	7b When applicable, explanation of any interim analyses and stopping guidelines	Not applicable	
Randomisation:			
Sequence generation	8a	Method used to generate the random allocation sequence	P. 13
	8b	Type of randomisation; details of any restriction (such as blocking and block size)	P.13
Implementation9	10	P.13	
Allocation concealment mechanism	Who generated the random allocation sequ		

	ence, who enrolled participants, and who assigned participants to interventions		
Blinding	11a	If done, who was blinded after assignment to interventions (for example, participants, care providers, those assessing outcomes) and how	P.13
	11b	If relevant, description of the similarity of interventions	Not applicable
	12a	Statistical methods used to compare groups for primary and secondary outcomes	PP.15
	12b	Methods for additional analyses, such as subgroup analyses and adjusted analyses	Not applicable
Not applicable yetParticipant flow (a diagram is strongly recommended)			
	13b	For each group, losses and exclusions after randomisation, together with reasons	PP. 9, 10

13aFor each group, the numbers of participants who were randomly assigned, received intended treatment, and were analysed for the primary outcome	14a	Dates defining the periods of recruitment and follow-up	Not applicable yet
	14b	Why the trial ended or was stopped	Not applicable yet
	15	A table showing baseline demographic and clinical characteristics for each group	PP. 18-20
Numbers analysed	16	For each group, number of participants (denominator) included in each analysis and whether the analysis was by original assigned groups	Not applicable yet
Outcomes and estimation	17a	For each primary and secondary outcome, results for each group, and the estimated effect size and its precision (such as 95% confidence interval)	Not applicable Yet
	17b	For binary outcomes, presentation of both absolute and relative effect sizes is recommended	Not applicable Yet
	18	Results of any other analyses performed, including subgroup analyses and adjusted analyses, distinguishing pre-specified from exploratory	Not applicable yet
Harms	19	All important harms or unintended effects in each group (for specific guidance see CONSORT for harms)	Not applicable yet
**Discussion**			
P.15Limitations			
Generalisability20 Trial limitations, addressing sources of potential bias, imprecision, and, if relevant,	21	Generalisability (external validity, applicability) of the trial findings	Not applicable

multiplicity of analyses			
Interpretation	22	Interpretation consistent with results, balancing benefits and harms, and considering other relevant evidence	Not applicable yet
**Other information**			
Registration			23
Protocol*REGISTRATION NUMBER AND NAME OF TRIAL REGISTRYP.3*	24	Where the full trial protocol can be accessed, if available	Not available yet
Funding	25	Sources of funding and other support (such as supply of drugs), role of funders	P.22

*We strongly recommend reading this statement in conjunction with the CONSORT 2010 Explanation and Elaboration for important clarifications on all the items. If relevant, we also recommend reading CONSORT extensions for cluster randomised trials, non-inferiority and equivalence trials, non-pharmacological treatments, herbal interventions, and pragmatic trials. Additional extensions are forthcoming: for those and for up to date references relevant to this checklist, see www.consort-statement.org.
