# Supplementary material for: Rationale and Design of Khuzestan Vitamin D Deficiency Screening Program in Pregnancy: A Stratified Randomized Vitamin D Supplementation Controlled Trial
Source: JMIR Res Protoc. 2017 Apr 7;6(4):e54. doi: 10.2196/resprot.7159 (PMC5400884; doi:10.2196/resprot.7159)
Supplement: Multimedia Appendix 1 [file resprot_v6i4e54_app1.pdf]

Research Institute for Endocrine Sciences
Shaheed Beheshti University of Medical Sciences
WHO Collaborating Center
for Research and Training Endocrine Science

پژوهشکده علوم غدد درون ریز و متابولیسم
دانشگاه علوم پزشکی و خدمات بهداشتی درمانی شهید بهشتی
مرکز همکاریهای سازمان جهانی بهداشت
برای پژوهش و آموزش علوم غدد درون ریز و متابولیسم
قطب علمی پزشکی کشور

August10th, 2013

Research Institute for Endocrine Sciences and Metabolism

Shahid Beheshti University of Medical Sciences

24 Parvaneh, Yaman Street, Velenjak, P.O.Box:19395-4763, Tehran, I.R.Iran.

Postal Code: 1985717413

Subject: Feedback from peer-review of full application for proposal: entitled "Vitamin D supplementation in pregnant women and pregnancy outcomes".

Dear Dr. Ramezani Tehrani

Thank you for submitting your Proposal. Your application has been assessed by internal and external reviewers and I enclose their comments for your attention. Please respond to any major issues and questions raised and submit your responses as an attachment (pdf Format) by 10 Sep 2013.

Thank you in advance for your cooperation and I look forward to receiving your response in due course.

Sincerely,

Dr. Azita Zadeh Vakilli A. Zadeh-Vakili

Deputy Director for Research

Reviewer 1:

Major:

1-What was your assumption for calculating the sample size in 2nd phase of the study? I suggest to recalculate it using some important pregnancy outcomes rather than cord vitamin D. please add more detail

2- the study procedure, random allocation and drug adherence have not been well described, please add more details.

2- Is it ethical that participants of Shooshtar not receiving Vitamin D?

Minor:

1-Please describe what you mean by moderate or severe vitamin D deficiency

2-please add a practical definition for each adverse outcome e.g GDM, Preterm,..

3- How long do you follow their babies?

4-please add inform consent.

Reviewer 2:

1- What was your assumption for sample size calculation? Cord vitamin D can not precisely identify the pregnancy outcomes, use some outcomes such as preterm delivery or..
2- Are these two cities are similar in term of nutritional habit, sun exposure,… if not this can highly affect your results
3- What are your assumed approaches for data analysis? Do you want to use intention to treatment analysis or other approach
4- What is your definition for sever or moderate vitamin D deficiency?

Reviewer 3

It is a well-designed study and if this research is successful, it will be highly usable. The only limitation of the research is that the number of participants in each arm of phase 2 is small which means some low prevalent pregnancy outcomes cannot be assessed. I suggest to add specific definition for each variable, e.g moderate vitamin D deficiency,… and also include inform consent.

Reviewer 4:

Major:

1-Are they two cities are comparable in term of sun exposure, vitamin D consumption and…

2- You mentioned that you are going to compare pregnancy outcomes however your calculated sample size are based on vitamin D concentration.

3- The study procedure has not been described well, how do you assess the drug adherence? Who are blind to study? What do you mean by moderate/sever vitamin D deficiency?

Minor: there are some typo error rechecked it

Add a figure to summarize the study procedure
