# Supplementary material for: Rationale and Design of Khuzestan Vitamin D Deficiency Screening Program in Pregnancy: A Stratified Randomized Vitamin D Supplementation Controlled Trial
Source: JMIR Res Protoc. 2017 Apr 7;6(4):e54. doi: 10.2196/resprot.7159 (PMC5400884; doi:10.2196/resprot.7159)
Supplement: Multimedia Appendix 2 [file resprot_v6i4e54_app2.pdf]

Subject: Re-Feedback from peer-review of full application for proposal: entitled “Vitamin D supplementation in pregnant women and pregnancy outcomes”.

Date:1 Sep 2013

Dear Dr. Zadeh Vakilli

Research director of Research Institute for Endocrine Sciences and metabolism

Please find our responses to the reviewers on the 14 pages that follow. We would like to thank the four reviewers for their constructive comments and we are grateful for the opportunity to respond.

Thank you for considering our application further.

Dr. Fahimeh Ramezani Tehrani

Principle investigator

Reviewer 1:

Major:

1-What was your assumption for calculating the sample size in 2nd phase of the study? I suggest to recalculate it using some important pregnancy outcomes rather than cord vitamin D. please add more detail.

Thank you for your valuable comment, we recalculate the sample size and used two assumptions, first one comparing the various protocols based on cord serum vitamin D and screening vs. no screening using pregnancy outcome. Here you can find the detail explanation for sample size calculation..

R1-A cluster sampling method, with PPS procedure will be used. Sample size is calculated in screening group (Masjed-Soleyman) using the following formula and assumption, resulting in 1537 subjects.

$$n \geq \frac{z_{1-\alpha/2}^{2}\left(1-P\right)}{\varepsilon^{2}P} \quad \begin{array}{l} \alpha = 0.05 \Rightarrow z_{1-\alpha/2} = 1.96 \\ P = 0.10 \\ \varepsilon = 0.15 \end{array}$$

The same steps (except for ε=0.2) will be used for calculation of sample size in no screening group (Shushtar), resulting in 900 subjects.

Since the prevalence of the specified event is untreated or unrecognized and the number of people who have the risk factor (P) is seemed to be high in the population (BMC Pregnancy Childbirth. 2007 Feb 12;7:1.Vitamin D status in mothers and their newborns in Iran), consequently, sample size needed for screening is considered sufficient. In other words, we

define a correction coefficient by (1/P) for estimated sample size (n), and then we expect n(1/P) number of people to find the 0.15 difference with 5% significance level.

To compare different regimens used for vitamin D deficiency in screening group, the sample size in each group is calculated based on the following formula and assumption:

$$n \geq \frac{\left(z_{\alpha/2} + z_{\beta}\right)^2 \sigma^2 \left(1 + 1/k\right)}{\varepsilon^2}$$

$$\alpha = 0.05 \Rightarrow z_{\alpha} = 1.96$$

$$1 - \beta = 0.90 \Rightarrow z_{\beta} = 1.28$$

$$\varepsilon = \mu_1 - \mu_2$$

$$k = 1$$

$$\theta = effect \quad size = |\varepsilon| / \sigma = 0.50$$

$$n = 2(1.96 + 1.28)^2 \left(\frac{1}{0.50}\right)^2 = 84$$

Considering loss to follow up of 10%, a total number of 100 in each study group will be considered adequate.

2- The study procedure, random allocation and drug adherence have not been well described, please add more details.

R2- All your requested information has been added and method section has been revised as follows. Furthermore a study flow chart has been added.

Subject Recruitment and Eligibility Criteria

Pregnant women, aged 18-40 years, are eligible if they had gestational age < 14 weeks based on last menstrual period or obstetrical estimation, singleton pregnancy and had planned to receive ongoing prenatal and delivery in the Masjed-Soleiman. Participants are excluded if they: consumed multi vitamins containing more than 400 IU/day of vitamin D3, used anticonvulsants and had history of chronic diseases like diabetes, hypertension, renal dysfunction, liver diseases and complicated medical or obstetrical history.

Data Collection and Quality Control Procedures

At first, midwives responsible for prenatal care in the selected health centers will be invited to attend a workshop designed for explaining the study objectives and procedure. Then, first trimester pregnant women seeking maternity care during their first routine prenatal visit are invited to participate in the study after providing a detailed explanation of the study procedure; they will signe a written informed consent at recruitment covering all trial procedures and data collection. At enrollment, for each participant a questionnaire that included information on socio-demographic, anthropomorphic, behavioral and reproductive characteristics was completed by a trained interviewer.

A fasting blood sample (5cc) will be taken by venipuncture from the antecubital vein. Then, plasma will separated by centrifugation (1500g, 10 min, 4˚C) in the central laboratory of Masjed-Soleyman and stored at -20˚C until analysis at end of the week. Serum concentrations of 25-hydroxyvitamin D for the participants of Masjed-Soleyman will be measured immediately and subsequently their vitamin D status will be determined. The samples in Shushtar laboratory will be stored at -80 C until end of the study; finally their serum concentration of vitamin D will be measured.

The Second Phase

In second phase of the study, Masjed-Soleyman participants will be assigned to screening program versus shushtar participants as non-screening arm. Within framework of the screening regimen, an eight arm blind randomized clinical trial was undertaken to compare the effects of various treatment protocols and

800 pregnant women with vitamin D deficiency from Masjed-Soleyman will be randomly allocated to one of the designed intervention programs according to the study flowchart.

Randomization and blinding

Subjects in each group of sever or moderate deficiencies will randomly divide into four subgroups using permuted block randomization by a biostatistician to achieve balance across treatment groups. The number of subjects per block was eight. Sealed opaque envelopes will be assigned to each subject by a research assistant not associated in the trial. The dedicated study midwife treating the females, who will not participate in any subsequent phases of the study, will be the only person who knew which group each patient belongs to (single blindness).

Adherence to medication regimen

Adherence to the supplementation regimen will be measured by maternal self-report and pill counts at each prenatal visit. The number of pills returned will be divided by the expected number of pills that would have been taken to create a percentage that indicates the adherence of medication regimen.

3- Is it ethical that participants of Shooshtar not receiving Vitamin D?

R3- We believe it is ethical, as screening of pregnant women for vitamin D deficiency has not been recommended yet. We tried to collect our blood samples at the same time that pregnant women referred for doing routine 1st prenatal lab.

Minor:

1-Please describe what you mean by moderate or severe vitamin D deficiency.

R1-severe deficient mean serum level of 25-hydroxyvitamin D (25(OH) D)<10ng/ml and moderate deficient mean serum level of 25-hydroxyvitamin D (25(OH) D)10-20ng/ml. It has been added to the text.

2-please add a practical definition for each adverse outcome e.g GDM, Preterm,…

R2-They have been added as follows:

Preterm delivery : birth at less than 37 completed weeks of gestation. Miscarriage: pregnancy loss prior to 20 weeks from LMP. Preeclampsia : systolic blood pressure >140 mmHg or diastolic blood pressure ≥90 mmHg and 24-hour proteinuria ≥0.3 g, started at >20 weeks. Premature rupture of membrane was considered as rupture of the fetal membranes before the onset of labor regardless of gestational age

APGAR score comprises five components including appearance, pulse, grimace, activity and respiration) each of which is given a score of 0-2 and reported at 1 and 5 minute after birth. Cord falling off time (day) is the separation time of remained umbilical cord after birth.

3- How long do you follow their babies?

R3-From delivery till discharge from hospital.

4-please add inform consent.

R4-Has been added.

Reviewer 2:

1- *What was your assumption for sample size calculation? Cord vitamin D can not precisely identify the pregnancy outcomes, use some outcomes such as preterm delivery or.*

R1- Thank you for your valuable comment, as we explained in response to reviewer 1, we revised it as follows:

R1-A cluster sampling method, with PPS procedure will be used. Sample size is calculated in screening group (Masjed-Soleyman) using the following formula and assumption, resulting in 1537 subjects.

$$n \geq \frac{z^2_{1-\alpha/2}(1-P)}{\varepsilon^2 P} \qquad \begin{aligned} &\alpha = 0.05 \Rightarrow z_{1-\alpha/2} = 1.96 \\ &P = 0.10 \\ &\varepsilon = 0.15 \end{aligned}$$

The same steps (except for ε=0.2) will be used for calculation of sample size in no screening group (Shushtar), resulting in 900 subjects.

Since the prevalence of the specified event is untreated or unrecognized and the number of people who have the risk factor (P) is seemed to be high in the population (BMC Pregnancy Childbirth. 2007 Feb 12;7:1.Vitamin D status in mothers and their newborns in Iran), consequently, sample size needed for screening is considered sufficient. In other words, we define a correction coefficient by (1/P) for estimated sample size (n), and then we expect n(1/P) number of people to find the 0.15 difference with 5% significance level.

To compare different regimens used for vitamin D deficiency in screening group, the sample size in each group is calculated based on the following formula and assumption:

$$n \geq \frac{(z_{\alpha/2} + z_\beta)^2 \sigma^2 (1 + 1/k)}{\varepsilon^2}$$

$$\alpha = 0.05 \Rightarrow z_\alpha = 1.96$$

$$1 - \beta = 0.90 \Rightarrow z_\beta = 1.28$$

$$\varepsilon = \mu_1 - \mu_2$$

$$k = 1$$

$$\theta = effect \quad size = |\varepsilon| / \sigma = 0.50$$

$$n = 2(1.96 + 1.28)^2 \left(\frac{1}{0.50} \right)^2 = 84$$

Considering loss to follow up of 10%, a total number of 100 in each study group will be considered adequate.

2- *Are these two cities are similar in term of nutritional habit, sun exposure,… if not this can highly affect your results*

R2- It seems that these two cities are highly similar, we added this part to justify our assumption:

Masjed-Soleyman County is in the northeast of Khuzestan province. Its area is 9/6327 km2 with a population of 103,369 people with Persian ethnicity. This is a sunny region with hot humid climate. Its altitude is 260 meters above sea level. In terms of geographical location, it is between 31°59′ E longitude and 49°17′ N latitude. Shushtar County is in the north of Khuzestan. Its area is 2436 km2 with a population of 192,361 people with Persian ethnicity. The climate is similar to Masjed-Soleyman. Its altitude is 150 meters above sea level. In terms of geographical location, it is between 48°20′ E longitude and 32°30′ N latitude.

3- *What are your assumed approaches for data analysis? Do you want to use intention to treatment analysis or other approach*.

R3- The detail of assumed data analysis has been added as follows. We will use intention to treatment analysis.

Statistical analysis:

Data will be expressed as mean± standard deviation (SD) for normally distributed variables and frequency (%) or median (IQR) (interquartile range) for categorical and/or non-normal variables. Continuous variables are checked for normality using the one sample Kolmogorov-Smirnov test. Distribution of variables between the two groups is compared using t-test or Mann-Whitney non-parametric test in case of normality violation and reported as mean± standard deviation. Categorical variables are compared using Pearson and Chi-squared tests. An intention to treat (ITT) analysis of the results is used. Generalized Linear Models will be used to assess the relationships between outcomes and exposures (here vitamin D3 supplementation intervention) adjusted by appropriate covariates. Analyses will be performed using the software version 19 (SPSS, Chicago, IL, USA). P-value of less than 0.05 is considered as significance level.

4- *What is your definition for sever or moderate vitamin D deficiency?*

R4-severe deficient mean serum level of 25-hydroxyvitamin D (25(OH) D)<10ng/ml and moderate deficient mean serum level of 25-hydroxyvitamin D (25(OH) D)10-20ng/ml. It has been added to the text.

Reviewer 3

It is a well-designed study and if this research is successful, it will be highly usable. The only limitation of the research is that the number of participants in each arm of phase 2 is small which means some low

prevalent pregnancy outcomes cannot be assessed. I suggest to add specific definition for each variable, e.g moderate vitamin D deficiency,… and also include inform consent.

Thank you for acknowledgment of our study, as you rightly mentioned we do not have adequate power for comparing the rare pregnancy outcomes such as abruption placenta, still birth and so on. However unfortunately due to time and budget limit we will not able to increase our sample size.

These definitions has been added.

Inform consent has been added to the research proposal.

Reviewer 4:

Major:

1-Are they two cities are comparable in term of sun exposure, vitamin D consumption and….

R1- as we mentioned in response to reviewer 2, we think these two cities are similar and we add some points to justify our assumption as follows:

Masjed-Soleyman County is in the northeast of Khuzestan province. Its area is 9/6327 km2 with a population of 103,369 people with Persian ethnicity. This is a sunny region with hot humid climate. Its altitude is 260 meters above sea level. In terms of geographical location, it is between 31°59′ E longitude and 49°17′ N latitude. Shushtar County is in the north of Khuzestan. Its area is 2436 km2 with a population of 192,361 people with Persian ethnicity. The climate is similar to Masjed-Soleyman. Its altitude is 150 meters above sea level. In terms of geographical location, it is between 48°20′ E longitude and 32°30′ N latitude.

2- You mentioned that you are going to compare pregnancy outcomes however your calculated sample size are based on vitamin D concentration.

As we mentioned in response to reviewers 1 and 2, we revised this part as follows:

A cluster sampling method, with PPS procedure will be used. Sample size is calculated in screening group (Masjed-Soleyman) using the following formula and assumption, resulting in 1537 subjects.

$$n \geq \frac{z_{1-\alpha/2}^2 (1-P)}{\varepsilon^2 P} \quad \begin{array}{l} \alpha = 0.05 \Rightarrow z_{1-\alpha/2} = 1.96 \\ P = 0.10 \\ \varepsilon = 0.15 \end{array}$$

The same steps (except for ε=0.2) will be used for calculation of sample size in no screening group (Shushtar), resulting in 900 subjects.

Since the prevalence of the specified event is untreated or unrecognized and the number of people who have the risk factor (P) is seemed to be high in the population (BMC Pregnancy Childbirth. 2007 Feb 12;7:1.Vitamin D status in mothers and their newborns in Iran), consequently, sample size needed for screening is considered sufficient. In other words, we define a correction coefficient by (1/P) for estimated sample size (n), and then we expect n(1/P) number of people to find the 0.15 difference with 5% significance level.

To compare different regimens used for vitamin D deficiency in screening group, the sample size in each group is calculated based on the following formula and assumption:

$$n \geq \frac{(z_{\alpha/2} + z_\beta)^2 \sigma^2 (1 + 1/k)}{\varepsilon^2}$$

$$\alpha = 0.05 \Rightarrow z_\alpha = 1.96$$

$$1-\beta = 0.90 \Rightarrow z_\beta = 1.28$$

$$\varepsilon = \mu_1 - \mu_2$$

$$k = 1$$

$$\theta = effect \quad size = |\varepsilon| / \sigma = 0.50$$

$$n = 2(1.96 + 1.28)^2 \left(\frac{1}{0.50} \right)^2 = 84$$

Considering loss to follow up of 10%, a total number of 100 in each study group will be considered adequate.

3- The study procedure has not been described well, how do you assess the drug adherence? Who are blind to study? What do you mean by moderate/sever vitamin D deficiency?

R3- your requested information has been added as follows:

Subject Recruitment and Eligibility Criteria

Pregnant women, aged 18-40 years, are eligible if they had gestational age < 14 weeks based on last menstrual period or obstetrical estimation, singleton pregnancy and had planned to receive ongoing prenatal and delivery in the Masjed-Soleiman. Participants are excluded if they: consumed multi vitamins containing more than 400 IU/day of vitamin D3, used anticonvulsants and had history of chronic diseases like diabetes, hypertension, renal dysfunction, liver diseases and complicated medical or obstetrical history.

Data Collection and Quality Control Procedures

At first, midwives responsible for prenatal care in the selected health centers will be invited to attend a workshop designed for explaining the study objectives and procedure. Then, first trimester pregnant women seeking maternity care during their first routine prenatal visit are invited to participate in the study after providing a detailed explanation of the study procedure; they will signe a written informed consent

at recruitment covering all trial procedures and data collection. At enrollment, for each participant a questionnaire that included information on socio-demographic, anthropomorphic, behavioral and reproductive characteristics was completed by a trained interviewer.

A fasting blood sample (5cc) will be taken by venipuncture from the antecubital vein. Then, plasma will separated by centrifugation (1500g, 10 min, 4˚C) in the central laboratory of Masjed-Soleyman and stored at -20˚C until analysis at end of the week. Serum concentrations of 25-hydroxyvitamin D for the participants of Masjed-Soleyman will be measured immediately and subsequently their vitamin D status will be determined. The samples in Shushtar laboratory will be stored at -80 C until end of the study; finally their serum concentration of vitamin D will be measured.

The Second Phase

In second phase of the study, Masjed-Soleyman participants will be assigned to screening program versus shushtar participants as non-screening arm. Within framework of the screening regimen, an eight arm blind randomized clinical trial was undertaken to compare the effects of various treatment protocols and 800 pregnant women with vitamin D deficiency from Masjed-Soleyman will be randomly allocated to one of the designed intervention programs according to the study flowchart.

Randomization and blinding

Subjects in each group of sever or moderate deficiencies will randomly divide into four subgroups using permuted block randomization by a biostatistician to achieve balance across treatment groups. The number of subjects per block was eight. Sealed opaque envelopes will be assigned to each subject by a research assistant not associated in the trial. The dedicated study midwife treating the females, who will not participate in any subsequent phases of the study, will be the only person who knew which group each patient belongs to (single blindness).

Adherence to medication regimen

Adherence to the supplementation regimen will be measured by maternal self-report and pill counts at each prenatal visit. The number of pills returned will be divided by the expected number of pills that would have been taken to create a percentage that indicates the adherence of medication regimen.

Severe deficient mean serum level of 25-hydroxyvitamin D (25(OH) D)<10ng/ml and moderate deficient mean serum level of 25-hydroxyvitamin D (25(OH) D)10-20ng/ml.

Minor: there are some typo error rechecked it.

R-We re-checked at corrected.

Add a figure to summarize the study procedure.

R-Thank you for your valuable suggestion, we added the study follow chart.
