# Supplementary material for: Rationale and Design of Khuzestan Vitamin D Deficiency Screening Program in Pregnancy: A Stratified Randomized Vitamin D Supplementation Controlled Trial
Source: JMIR Res Protoc. 2017 Apr 7;6(4):e54. doi: 10.2196/resprot.7159 (PMC5400884; doi:10.2196/resprot.7159)
Supplement: Multimedia Appendix 3 [file resprot_v6i4e54_app3.pdf]

January 10, 2014th

Research Institute for Endocrine Sciences

Shahid Beheshti University of Medical Sciences and Metabolism

24 Parvaneh, Yaman Street, Velenjak, P.O.Box:19395-4763, Tehran, I.R.Iran.

Postal Code: 1985717413

Dear Dr. Ramezani Tehrani

We are pleased to inform you that your proposal entitled "Vitamin D supplementation in pregnant women and pregnancy outcomes" has been selected for funding at 202nd research council meeting of research Institute for Endocrine Sciences and Metabolism.

We look forward to working with you in the years ahead to shepherd this outstanding project to completion.

Sincerely,

Dr. Azita Zadeh Vakilli A. Zadeh Vakili

Deputy Director for Research
